# Differential Effects of Household Thermal Processing on Extractable β-Glucan and Total Resistant Starch Across Processed Barley Forms

**DOI:** 10.3390/foods15173089

**Published:** 2026-08-31

**Authors:** Shrunga Shree Shivanand, Prasanthi Prabhakaran Sobhana, Srinivas Epparapally, Vinay Kumar Soma, Raghavendra Rao Chowdavarapu, Gobeeswaran Sundaralingam, Sangita Thenarangam, Shally Vishnoi, Pavithra Rajakumar Chithra, Prathyusha Vasireddy, Radhika Madhari

**Affiliations:** 1 Department of Dietetics, ICMR–National Institute of Nutrition, Hyderabad 500007, India; shrungadafedar007@gmail.com (S.S.S.); sobhana.pp@icmr.gov.in (P.P.S.); epparapally.s@icmr.gov.in (S.E.); gobeeswaran.s@icmr.gov.in (G.S.); sangitathena@gmail.com (S.T.); vishnoishally4@gmail.com (S.V.); rcpavithraj@gmail.com (P.R.C.); prathyushavasireddy05@gmail.com (P.V.); 2 Clinical Epidemiology Division, ICMR–National Institute of Nutrition, Hyderabad 500007, India; vinaysoma100@gmail.com; 3 Maternal and Child Health Nutrition (MCHN) Division, ICMR–National Institute of Nutrition, Hyderabad 500007, India

**Keywords:** barley forms, extractable β-glucan, total resistant starch, household thermal processing, pressure cooking, microwave cooking, boiling, roasting, functional foods, food matrix

## Abstract

Barley (*Hordeum vulgare* L.) is rich in β-glucan and resistant starch, key functional carbohydrates that are determinants of metabolic health. However, evidence on the effects of household processing on these fractions across barley forms remains limited. This study evaluated soaking and four household thermal processing methods (pressure cooking, microwave cooking, boiling, and dry roasting) on extractable β-glucan and total resistant starch in pearled barley, barley grits and barley flour. Pressure cooking produced the highest observed extractable β-glucan in pearled barley (6.40 ± 0.22 vs. raw 4.24 ± 0.17 g/100 g) and grits (5.80 ± 0.17 vs. raw 5.31 ± 0.27 g/100 g), whereas boiling was associated with highest extractability in flour (5.20 ± 0.17 vs. raw 4.59 ± 0.23 g/100 g). Soaked pressure cooking resulted in greater total resistant starch content in pearled barley (8.02 ± 0.11 vs. raw 5.24 ± 0.04 g/100 g) and grits (5.33 ± 0.09 vs. raw 2.10 ± 0.13 g/100 g), while non-soaked boiling showed a greater response in flour (1.07 ± 0.04 vs. raw 0.22 ± 0.01 g/100 g). The exploratory findings suggest that barley form may influence the response of functional carbohydrates to household thermal processing. Translating these outcomes into metabolic benefits requires further physicochemical, metabolic, and physiological validation.

## 1. Introduction

The increasing global burden of chronic metabolic disorders has intensified interest in food-based strategies for their prevention and management, thereby increasing the scientific and commercial importance of functional foods containing naturally occurring bioactive constituents [1,2]. Among cereal grains, barley (*Hordeum vulgare* L.) has attracted considerable attention because of its favourable nutritional profile and its potential role in supporting healthy glucose metabolism, lipid homeostasis and gastrointestinal function [2,3,4]. Consequently, barley is increasingly incorporated into a wide range of food products intended for health-conscious consumers and populations at increased metabolic risk [2,3].

The health-promoting properties of barley arise from the combined contribution of several bioactive constituents, including soluble dietary fibre, resistant starch, phenolic compounds, phytosterols and tocols [2,4]. Among these, β-glucan and resistant starch are the two most extensively investigated functional carbohydrate fractions because of the substantial evidence supporting their physiological relevance [3,5]. β-Glucan, a soluble mixed-linkage polysaccharide present in relatively high concentrations within barley cell walls, increases intestinal viscosity, thereby attenuating postprandial glycaemic responses and reducing circulating cholesterol concentrations [3,6,7]. These physiological benefits have been recognised by regulatory authorities, including the United States Food and Drug Administration and the European Food Safety Authority, which have approved specific health claims relating to barley β-glucan [8,9]. In contrast, resistant starch escapes digestion in the small intestine and undergoes fermentation by the colonic microbiota, producing short-chain fatty acids that contribute to gut health and metabolic regulation [5]. Although these functional carbohydrate fractions exert their physiological effects through distinct mechanisms, together they represent key functional carbohydrate components contributing to the nutritional and physiological value of barley [2,3,5].

The compositional characteristics of cereal grains, however, do not remain static during food preparation. Household processing influences grain hydration, starch gelatinization, molecular interactions and the accessibility of food constituents, thereby modifying both the extractability and the functional properties of cereal carbohydrate fractions [3,10,11,12]. Consequently, the nutritional value of barley-based foods depends not only on the intrinsic composition of the grain but also on the manner in which it is prepared before consumption [3,11]. Understanding these processing-induced compositional changes is therefore essential for developing household processing recommendations that can improve the functional carbohydrate profile of barley-based foods.

The influence of household processing is further complicated by the diversity of commercially available barley forms. Pearled barley, barley grits and barley flour differ substantially in particle size, degree of structural disruption and physical integrity, characteristics that influence hydration behaviour, heat transfer and starch transformations during cooking [13,14]. These structural differences are expected to modify the responses of extractable β-glucan and total resistant starch to household processing [3,5]. The food matrix concept provides a useful framework for interpreting these differential responses because the structural organization of cereal tissues governs hydration behaviour, heat transfer and the accessibility of carbohydrate polymers during household processing [15,16,17]. Consequently, identical household processing methods may not necessarily produce equivalent compositional outcomes across different barley forms.

Despite increasing interest in barley as a functional cereal, relatively few studies have systematically evaluated how commonly employed household processing practices influence multiple functional carbohydrate fractions across different commercially available barley forms under standardized conditions [2,3]. Previous investigations have demonstrated that milling, germination, extrusion, roasting, steaming and other thermal treatments can influence β-glucan extractability and total resistant starch content in barley [11,18,19,20,21]. However, most available studies have examined individual processing techniques or single barley products, thereby limiting direct comparisons of household processing responses across commonly consumed barley forms [11,18]. Furthermore, relatively little information is available regarding the combined influence of soaking and commonly employed household thermal processing methods under standardized experimental conditions [20,21]. Consequently, the basis for household processing recommendations for different barley forms remains limited.

We hypothesized that commonly employed household thermal processing methods would elicit differential responses in extractable β-glucan and total resistant starch according to the barley form. Therefore, the objective of the present study was to systematically evaluate the effects of soaking and four commonly employed household thermal processing methods, namely pressure cooking, microwave cooking, boiling and dry roasting, on extractable β-glucan and total resistant starch in pearled barley, barley grits and barley flour. This study also aimed to generate exploratory data on the household processing methods with potential for improving the functional carbohydrate profile of commercially available barley forms and to provide a scientific foundation for future investigations examining the physicochemical, physiological and metabolic significance of processing-induced compositional changes.

## 2. Materials and Methods

### 2.1. Experimental Design

This study employed a two-factor factorial experimental design to evaluate the effects of soaking and household thermal processing on extractable β-glucan and total resistant starch in three commercially available barley forms. Pearled barley, barley grits and barley flour were selected to represent products differing in particle size and degree of structural integrity. The experimental factors comprised soaking condition (non-soaked and soaked) and household thermal processing methods (pressure cooking, microwave cooking, boiling and dry roasting), while the unprocessed raw samples served as the reference for comparison. Following processing, all samples were analysed for moisture content, extractable β-glucan and total resistant starch using validated enzymatic methods, and all analytical results were expressed on a dry-weight basis after moisture correction.

In addition to the processed treatments, unprocessed raw samples of each barley form were analysed as baseline references. As an exploratory study, the factorial experimental design evaluated the combined effects of soaking and subsequent household thermal processing; therefore, raw barley samples were analysed only in their unsoaked state. Soaking is a preparatory step rather than a consumable household preparation method and under normal household practice, it is followed by household thermal processing before consumption. Consequently, a soaked-raw treatment was not included in the experimental design.

### 2.2. Procurement and Preparation of Barley Samples

Three barley forms, namely pearled barley, barley grits and barley flour, were procured from retail and wholesale markets located across three geographically distinct zones of Hyderabad, India. These barley forms were selected because they represent the principal commercially available forms differing in particle size and degree of structural integrity. Within each zone, samples were obtained from wholesale markets and retail outlets, including supermarkets and neighbourhood grocery stores, to improve the representativeness of commercially available products. A total of nine individual samples, comprising three samples from each market type, were collected for each barley form.

**Figure 1:**
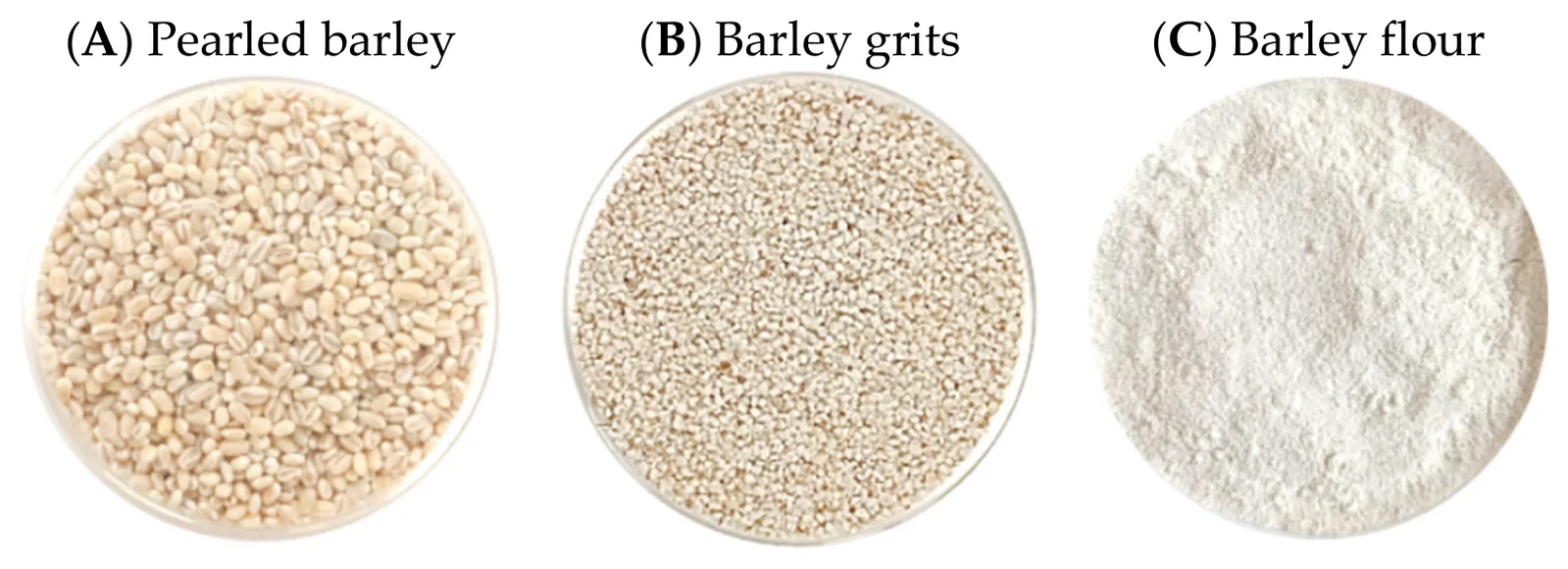
Representative images of the three commercially available barley forms used for household processing and compositional analyses, which depict distinct physical characteristics in raw form: (**A**) pearled barley, intact grain structure; (**B**) barley grits, coarse grain particles of intermediate size; and (**C**) barley flour, a finely milled powder. Composite samples were prepared by pooling representative samples collected from retail and wholesale commercial outlets before soaking, household thermal processing and compositional analyses.

Visible foreign materials were removed manually prior to analysis. Individual samples belonging to each barley form were pooled to prepare representative composite samples, thereby minimizing variation attributable to differences among individual commercial sources. The composite samples were milled to pass through a 0.5 mm sieve, homogenized thoroughly and stored in airtight containers at 4 °C until further analysis. Representative images of the three barley forms are shown in Figure 1.

### 2.3. Standardization of Household Pre-Treatment and Household Thermal Processing Conditions

Preliminary standardization experiments were conducted prior to the commencement of the study to establish reproducible household thermal processing conditions for each barley form. The objective of the standardization was to determine the minimum water volume and processing duration required to achieve predefined cooking endpoints while avoiding under-processing or excessive cooking.

Because pearled barley, barley grits and barley flour differ substantially in particle size, degree of structural integrity and hydration behaviour, separate processing conditions were established for each barley form. Samples were evaluated under two pre-processing conditions, namely non-soaking and soaking. Based on preliminary hydration studies, the soaking duration was standardized to 15 h for pearled barley and 2 h for both barley grits and barley flour. The longer soaking period for pearled barley reflects its relatively intact grain structure and slower rate of water absorption, whereas the greater exposed surface area of barley grits and barley flour facilitated more rapid hydration. A soaking duration of 2 h was applied for barley grits and barley flour, reflecting their practical relevance to common household preparation practices. For barley grits and pearled barley, adequate hydration was confirmed by the absence of a perceivable hard core upon gentle compression. In the case of barley flour, due to mixing with the soaking medium, a qualitative indicator of hydration was not applicable; consequently, a uniform soaking time of 2 h was adopted.

Standardized processing endpoints were established a priori during the standardization phase to ensure consistency across all experimental treatments. For pearled barley, household thermal processing was considered complete when the kernels were fully hydrated, as indicated by the absence of a hard opaque core on manual sectioning. For barley grits, the endpoint was defined as complete hydration with no perceptible hard centre upon gentle compression. For barley flour, processing was considered complete when a homogeneous gelatinized paste was obtained without visible ungelatinized flour particles. Dry roasting was terminated when the samples developed a uniform light brown colour accompanied by the characteristic aroma of evenly roasted barley.

All household thermal processing treatments were performed according to standardized protocols using predetermined equipment, sample-to-water ratios, processing times and operating conditions specific to each barley form and cooking method. Following household thermal processing, samples were allowed to reach ambient room temperature (30–32 °C) over 30 min, without controlled or forced cooling. This approach was intended to replicate the natural cooling conditions typical of household cooking practices. Samples were subsequently homogenized thoroughly, dried, milled and stored under standardized conditions prior to compositional analysis. The total resistant starch content was determined immediately once the thermally processed samples attained room temperature. The complete processing protocol for each treatment is summarized in Table 1.

The four household thermal processing methods selected, i.e., pressure cooking, microwave cooking, boiling and dry roasting, represent commonly employed household preparation techniques for cereal-based foods and were chosen to enhance the practical relevance of the findings.

### 2.4. Thermal Processing Procedures

Following pre-processing, samples were subjected to four standardized household thermal processing methods, namely pressure cooking, microwave cooking, boiling and dry roasting, using the processing conditions established during the preliminary standardization phase (Table 1). Processing parameters, including equipment, sample-to-water ratios, processing durations and operating conditions, were specific to each barley form and cooking method. Ultrapure water (18.2 MΩ·cm; Crystal EX, Adrona SIA, Riga, Latvia) was used for all moist-heat processing methods.

Pressure cooking was performed using a domestic stainless steel pressure cooker (TTK Prestige Ltd., Bengaluru, India). Microwave cooking was carried out in a 900 W microwave oven (LG Electronics India Ltd., Greater Noida, India) using borosilicate microwave-safe containers. Boiling and dry roasting were performed in tri-ply stainless steel vessels (GreenChef Appliances Ltd., Bengaluru, India), with boiling conducted under closed-lid conditions and roasting carried out with continuous stirring to ensure uniform heat distribution.

Each treatment combination (barley form × soaking condition × cooking method) was processed on a single occasion, using an aliquot of the composite pooled sample described in Section 2.2. Technical replicates were obtained by drawing multiple sub-aliquots from this single processed batch: four for extractable β-glucan (*n* = 4) and three for total resistant starch (*n* = 3).

Following completion of each household thermal processing treatment, samples were cooled to room temperature (30–32 °C), homogenized thoroughly and milled where necessary to obtain a uniform analytical sample. Raw samples were subjected to identical milling and homogenization procedures prior to analysis to ensure consistency in sample preparation across all treatments.

Moisture content values for all barley forms, treatments and replicates are provided in full as Supplementary Table S1, offering an objective quantitative reference along with the processing parameters summarized in Table 1.

The processed samples obtained following the standardized household thermal processing procedures were subsequently analysed for moisture content, extractable β-glucan and total resistant starch using validated analytical methods.

### 2.5. Moisture Determination

Moisture content was determined gravimetrically according to AOAC Official Method 925.10 [22] using duplicate determinations for each treatment. Approximately 2 g of each homogenized sample was dried at 105 °C to constant weight, and moisture content was calculated gravimetrically. Moisture values were subsequently used to express extractable β-glucan and total resistant starch concentrations on a dry-weight basis, thereby facilitating valid comparisons among barley forms and household thermal processing treatments.

### 2.6. Determination of Extractable β-Glucan

Extractable β-glucan was quantified using AOAC Official Method 995.16, employing the Mixed-Linkage β-Glucan Assay Kit (K-BGLU, Megazyme International Ireland Ltd., Bray, Ireland), following the manufacturer’s instructions without modification and the validated enzymatic procedure described by McCleary and Codd [20,21,23]. The assay is based on the enzymatic hydrolysis of mixed-linkage (1→3), (1→4)-β-D-glucan to glucose, which is subsequently quantified using a glucose oxidase/peroxidase (GOPOD) colorimetric reaction [20].

Briefly, appropriately prepared processed barley samples were subjected to standardized enzymatic extraction, followed by sequential hydrolysis with lichenase and β-glucosidase to release glucose from mixed-linkage β-glucan. The liberated glucose was quantified colorimetrically using the GOPOD reagent, and absorbance was measured at 510 nm using a Thermo Scientific™ Multiskan™ GO Microplate Spectrophotometer (Thermo Fisher Scientific, Waltham, MA, USA). Extractable β-glucan concentrations were calculated according to the manufacturer’s prescribed calculation procedure using appropriate reagent blanks and correction factors [20].

Throughout this manuscript, the term *extractable β-glucan* refers to β-glucan quantified using this standardized enzymatic procedure following the respective household thermal processing treatments. This terminology is used to distinguish β-glucan quantified using the standardized analytical procedure following processing from the total intrinsic β-glucan naturally present in barley, recognising that household thermal processing may modify extractability without altering the absolute β-glucan content of the grain.

Four technical extractions were performed from separately weighed aliquots of each homogenized processed sample, and each extract was analysed according to the standardized assay procedure. Results were expressed as g/100 g dry weight following moisture correction.

### 2.7. Determination of Total Resistant Starch

Total resistant starch was quantified using AOAC Official Method 2002.02, employing the Digestible and Resistant Starch Assay Kit (K-DSTRS, Megazyme International Ireland Ltd., Bray, Ireland), following the manufacturer’s instructions without modification. This analytical procedure is based on the selective enzymatic hydrolysis of digestible starch, followed by recovery and quantification of the total resistant starch, according to the validated enzymatic method [24,25].

Briefly, appropriately prepared processed barley samples were subjected to controlled enzymatic digestion using pancreatic α-amylase and amyloglucosidase to remove digestible starch under standardized incubation conditions. The total resistant starch remaining after enzymatic digestion was recovered, solubilized, hydrolysed to glucose and quantified using the glucose oxidase/peroxidase (GOPOD) colorimetric reaction. Absorbance was measured at 510 nm using a Thermo Scientific™ Multiskan™ GO Microplate Spectrophotometer (Thermo Fisher Scientific, Waltham, MA, USA), and total resistant starch concentrations were calculated according to the manufacturer’s prescribed calculation procedure using appropriate reagent blanks and correction factors [26].

Throughout this manuscript, the term *total resistant starch* refers to the starch fraction quantified using this standardized enzymatic procedure following the respective household thermal processing treatments. The analytical method estimates the fraction of starch resistant to enzymatic digestion under standardized in vitro conditions and provides an operational measure of total resistant starch available for comparative evaluation among the different household processing treatments.

Three technical extractions were performed from separately weighed aliquots of each homogenized processed sample, and each extract was analysed according to the standardized assay procedure. Results were expressed as g/100 g dry weight following moisture correction.

### 2.8. Outcome Measures

The primary outcome measures were the concentrations of extractable β-glucan and total resistant starch following standardized household thermal processing. Comparisons were undertaken across three barley forms (pearled barley, barley grits and barley flour), two pre-processing conditions (non-soaked and soaked) and four household thermal processing methods (pressure cooking, microwave cooking, boiling and dry roasting). Additionally, exploratory statistical analyses were conducted to evaluate differential processing responses among barley forms and to provide preliminary evidence to inform future household processing studies.

### 2.9. Statistical Analysis

Statistical analyses were performed using IBM SPSS Statistics (Version 23.0; IBM Corp., Armonk, NY, USA) [27]. Descriptive data are presented as means ± standard deviation (SD) with their 95% Confidence Intervals (95% CI). Distribution of data points are represented in Supplementary Figure S1. An exploratory analytical framework based on two-way factorial ANOVA was adopted. The assumptions of factorial ANOVA were evaluated using the Shapiro–Wilk test for normality and Levene’s test for homogeneity of variances, and full test statistics and *p*-values are provided in Supplementary Table S2. The balanced factorial design is considered robust to modest departures from these assumptions; therefore, the findings are interpreted as exploratory.

The effects of soaking (non-soaked and soaked) and household thermal processing method (pressure cooking, microwave cooking, boiling and dry roasting) on extractable β-glucan and total resistant starch were evaluated separately for each barley form using two-way factorial analysis of variance (ANOVA). The main effects of soaking and household thermal processing method, together with their interaction (soaking × household thermal processing method), were examined. In addition, partial eta squared (η^2^p) was calculated as an exploratory estimate of effect magnitude.

Raw samples served as baseline reference values and were not included in the exploratory factorial ANOVA because the experimental design evaluated the combined effects of soaking and subsequent household thermal processing. Raw samples were analysed only in the unsoaked state because soaking alone does not represent a consumable household preparation method and would require subsequent cooking. Each thermally processed treatment combination was compared with its corresponding raw sample using Dunnett’s post hoc test. Bonferroni-adjusted pairwise comparisons were used to compare household processing methods within each soaking condition of each barley form and did not include raw sample values.

Statistical significance was accepted at *p* < 0.05. Heat maps and Supplementary Figure S1 were generated using the R software (Version 4.6.0; R Foundation for Statistical Computing, Vienna, Austria) [28]. The heat maps were generated to visualize the percentage change in extractable β-glucan and total resistant starch relative to the corresponding raw samples for each barley form, soaking condition and household thermal processing method. Statistically significant increases relative to the raw samples are represented by shades of green, statistically significant decreases by shades of red, and non-significant changes by a neutral colour.

## 3. Results

### 3.1. Baseline Compositional Differences Among Processed Barley Forms

Before household thermal processing, the three commercially available barley forms differed in their baseline concentrations of extractable β-glucan and total resistant starch (Table 2 and Table 3). Extractable β-glucan was highest in barley grits (5.31 ± 0.27 g/100 g dry weight), followed by barley flour (4.59 ± 0.23 g/100 g) and pearled barley (4.24 ± 0.17 g/100 g). In contrast, total resistant starch was highest in pearled barley (5.24 ± 0.04 g/100 g dry weight), intermediate in barley grits (2.10 ± 0.13 g/100 g) and lowest in barley flour (0.22 ± 0.01 g/100 g). These baseline differences indicate that commercially available barley forms possess distinct functional carbohydrate profiles prior to household processing and therefore cannot be regarded as nutritionally equivalent. Accordingly, subsequent processing responses were interpreted relative to the corresponding raw sample for each barley form.

### 3.2. Effects of Soaking and Household Thermal Processing on Extractable β-Glucan

Distinct processing response patterns were observed across the three barley forms (Table 2). In the exploratory ANOVA, significant main effects of soaking and household thermal processing were observed in all three barley forms, whereas the soaking × household thermal processing interaction was significant in pearled barley and barley flour but not in barley grits (Supplementary Table S3).

**Figure 2:**
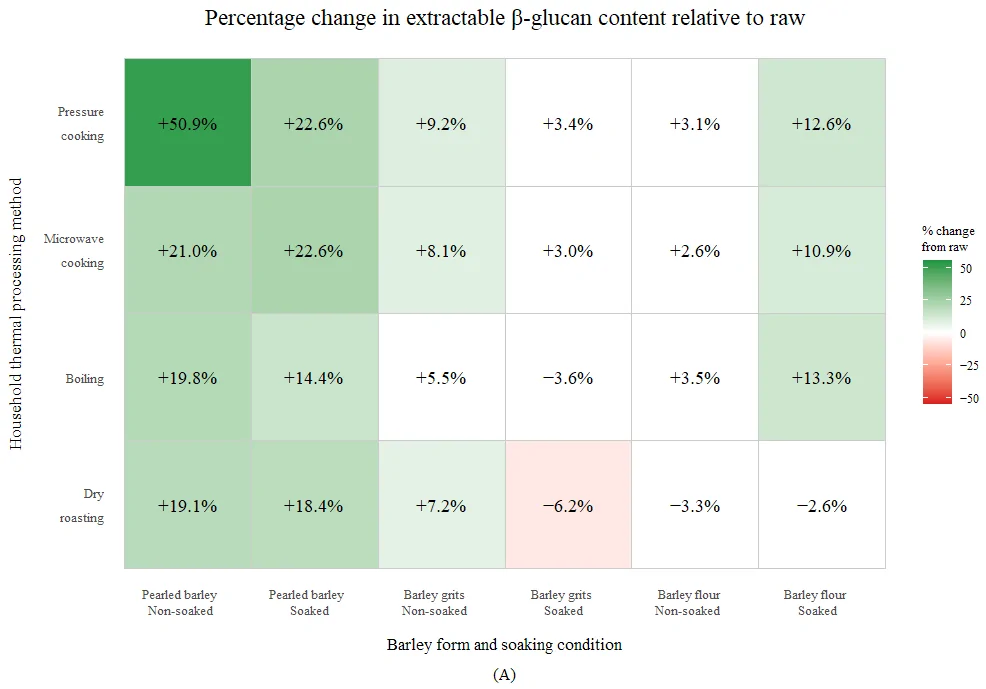

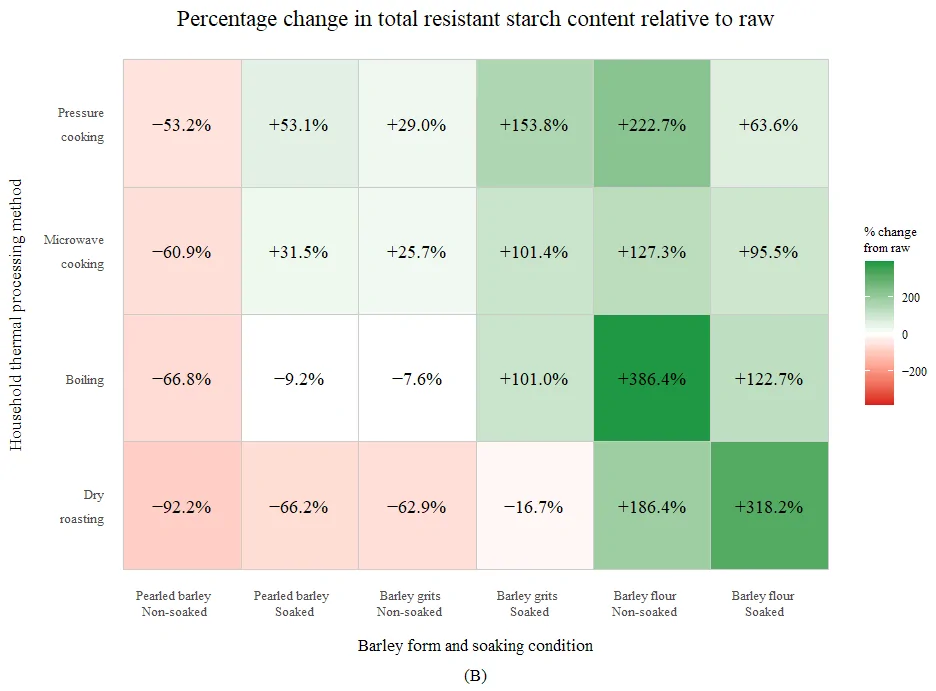
Heat map summarizing the effects of pre-treatment and household thermal processing on extractable β-glucan (**A**) and total resistant starch (**B**) across three barley forms. Values represent mean percentage change calculated as (processed mean value - raw mean value)/raw mean value × 100 using the data presented in Table 2 and Table 3. The colours green (increase) and red (decrease) are informed by statistically significant changes from raw reference, and no significant changes are retained as neutral (Dunnett’s test, *p* ≥ 0.05). The increase in colour intensity is in accordance with the increase in the magnitude of percentage change (increase in magnitude of positive change; increasing intensity of green; increase in magnitude of negative change; increasing intensity of red).

In pearled barley, pressure cooking without soaking was associated with the highest extractability of β-glucan content (6.40 ± 0.22 g/100 g dry weight), representing a significant increase relative to the raw reference. Microwave cooking, boiling and dry roasting were also associated with higher extractable β-glucan than the raw sample, although to a lesser extent than pressure cooking. This pattern is also reflected in the percentage heat map in Figure 2A. Following soaking, all household thermal processing methods maintained extractable β-glucan contents above the raw reference, with pressure cooking and microwave cooking producing the highest observed values (+22.6%).

Barley grits exhibited a different response pattern. Under non-soaked conditions, pressure cooking, microwave cooking and dry roasting produced similarly higher extractable β-glucan contents, whereas boiling yielded values comparable to the raw reference. Following soaking, while none of the methods improved extractability significantly, pressure cooking and microwave cooking maintained higher extractable β-glucan contents than boiling and dry roasting, and the latter resulted in a significant reduction relative to the raw content (−6.2%) (Figure 2A).

Barley flour exhibited a distinct processing response pattern from those observed in pearled barley and barley grits. Under non-soaked conditions, extractable β-glucan remained comparable to the raw reference following pressure cooking, microwave cooking and boiling. Although, dry roasting resulted in lower values, they still remained comparable to raw content. Under soaking conditions, boiling yielded the highest extractable β-glucan content (5.20 ± 0.17 g/100 g dry weight), closely followed by pressure cooking and microwave cooking, all of which were significantly greater than the corresponding raw sample (Figure 2A). Dry roasting, however, did not differ significantly from the raw reference.

Effect-size analysis indicated that both soaking and household thermal processing exerted large effects on extractable β-glucan, although household thermal processing generally showed larger effect sizes than soaking, except in barley grits (η^2^ = 0.435 for cooking method vs. 0.660 for soaking; Supplementary Table S4). However, these results are exploratory and are derived from a small sample size, although the observed pattern of effects provides preliminary evidence for form-specific processing responses. This study used technical replicates processed from a single batch of thermally processed samples per treatment, and the resulting variations reflect analytical variability. The subsequent F-statistics and effect sizes represent the differences within the analysed samples and not across independent processing batches.

Overall, the responses of extractable β-glucan varied across each barley form. Pressure cooking was associated with the highest observed extractable β-glucan content in pearled barley, pressure cooking and microwave cooking produced comparable extractable β-glucan contents in barley grits, and boiling following soaking yielded the greatest extractable β-glucan in barley flour. These exploratory findings suggest that household thermal processing for preserving extractable β-glucan may need to be tailored according to the barley form rather than generalized across barley products, as further illustrated in the percentage-change heat map (Figure 2A).

### 3.3. Effects of Soaking and Household Thermal Processing on Total Resistant Starch

Distinct processing response patterns were observed for total resistant starch. Exploratory effect-size estimates were larger than those observed for extractable β-glucan (Supplementary Table S4). The exploratory ANOVA indicated significant main effects of soaking, household thermal processing and their interaction for total resistant starch across all three barley forms (Supplementary Table S3).

Although the total resistant starch content decreased significantly following household thermal processing in the non-soaked pearled barley group, pressure cooking was associated with the smallest observed reduction (−53.2%) among all the household thermal processing methods. In contrast, total resistant starch content increased significantly following household thermal processing in the soaked pearled barley group, with pressure cooking yielding the greatest value (8.02 ± 0.11 g/100 g dry weight, +53.1%). Microwave cooking also increased total resistant starch relative to the raw reference, whereas boiling and dry roasting produced lower responses. The magnitude was particularly observed to be greater following dry roasting of pearled barley under non-soaked conditions. Overall, pressure cooking was associated with the highest observed total resistant starch response in pearled barley (Table 3).

Barley grits exhibited a response similar to that observed in pearled barley. Pressure cooking was associated with the highest observed total resistant starch content under both soaking conditions, followed by microwave cooking, whereas boiling and dry roasting resulted in lower values. Although soaking influenced the magnitude of the response, pressure cooking consistently produced the greatest total resistant starch content in barley grits. The significant interaction suggests that the response to household thermal processing varied according to soaking status (Supplementary Table S3).

In contrast, barley flour exhibited a distinct processing response pattern. Under non-soaked conditions, boiling produced the highest total resistant starch content (1.07 ± 0.04 g/100 g), whereas dry roasting yielded the greatest value following soaking (0.92 ± 0.03 g/100 g).

Microwave cooking produced intermediate total resistant starch contents, whereas dry roasting was associated with the lowest values in pearled barley and barley grits, except in barley flour. The significant interaction indicates that the influence of household thermal processing on total resistant starch in barley flour depended on soaking status (Supplementary Table S3).

Effect-size estimates (partial η^2^) were larger than those observed for extractable β-glucan (η^2^ = 0.946–0.994 for total resistant starch vs. 0.231–0.836 for β-glucan), suggesting larger processing-associated effects for total resistant starch than for extractable β-glucan under the experimental conditions studied (Supplementary Table S4). Despite the small sample size, this pattern was observed consistently across all three barley forms, suggesting that total resistant starch may respond more strongly to household thermal processing than extractable β-glucan under the experimental conditions studied. Furthermore, the estimation of total resistant starch was done using technical replicates processed from a single batch per treatment, and the resulting variations reflect analytical variability. The subsequent F-statistics and effect sizes are representative of differences within the analysed samples and not independent processing batches.

Pressure cooking was associated with the highest observed total resistant starch content in pearled barley and barley grits, whereas barley flour exhibited a distinct processing profile in which the household processing method associated with the highest total resistant starch content differed according to soaking status. These barley-form-specific processing responses are summarized quantitatively in the heat map (Figure 2B), while the corresponding potential household processing strategies are presented in Table 4.

### 3.4. Summary of Processing Response Across Barley Forms

The observed processing responses differed across the three barley forms under identical household processing conditions. Both the magnitude and direction of the responses depended on the barley form and the incorporation of soaking prior to household thermal processing, suggesting that the observed household processing responses were influenced by the physical form of barley.

Pressure cooking was associated with the greatest potential to modify overall functional carbohydrate contents in pearled barley and barley grits, although the inclusion of soaking differentially influenced extractable β-glucan and total resistant starch. In contrast, barley flour exhibited a distinct processing profile, with boiling after soaking producing the greatest extractable β-glucan, while total resistant starch responses varied according to the combination of soaking and household thermal processing method.

Soaking associated response patterns were observed to vary across the barley forms. In pearled barley and barley grits, soaking primarily enhanced total resistant starch content in comparison to the magnitude of change observed in the non-soaking group, whereas its influence on extractable β-glucan was comparatively modest. In barley flour, however, soaking modified the effects of household thermal processing on both functional carbohydrate fractions, resulting in processing responses that differed from those observed in pearled barley and barley grits.

Overall, these exploratory findings suggest that the functional carbohydrate profile of commercially available barley forms can be modulated through appropriate household processing. Accordingly, any household processing suggestions may need to be tailored to the barley form rather than generalized across barley products. The integrated processing responses are summarized in the percentage-change heat map (Figure 2), while the corresponding potential household processing suggestions are presented in Table 4.

## 4. Discussion

### 4.1. Principal Exploratory Findings

The present study explored the effects of household processing on the functional carbohydrate composition of barley. The exploratory findings suggest that processing responses vary according to barley form and therefore cannot be generalized across commercially available barley products. Although identical household processing methods were applied under standardized conditions, distinct processing response patterns were observed for pearled barley, barley grits and barley flour. Furthermore, the inclusion of soaking modified these responses in a barley-form-specific manner, highlighting the importance of considering both barley form and the sequence of household processing steps when evaluating compositional changes. Collectively, these findings indicate that maximizing functional carbohydrate composition may require consideration of both household processing conditions and barley form, rather than assuming a uniform response across commercially available barley forms [15,29,30].

A notable finding of the present study is that extractable β-glucan and total resistant starch did not respond similarly to household processing. Extractable β-glucan generally exhibited comparatively modest processing-induced changes, whereas total resistant starch was observed to be highly sensitive to both soaking and household thermal processing, as indicated by the larger descriptive percentage changes and exploratory effect-size estimates. These observations suggest that the two functional carbohydrate fractions respond differently to household processing and therefore may require independent consideration when developing processing strategies [3,5,7,10,31]. However, it should be noted here that these results arise from a comparatively smaller sample size, and the inferences are preliminary.

Another notable finding is that no single household processing method consistently improved both functional carbohydrate fractions across all barley forms. Pressure cooking was associated with the highest overall increases for pearled barley and barley grits; barley flour exhibited a distinct processing profile in which boiling produced the greatest extractable β-glucan, and depending on soaking status, the total resistant starch content varied. These exploratory findings suggest that household processing recommendations should be tailored to the specific barley form rather than generalized across barley products.

In addition to identifying potential household thermal processing approaches, the present study provides an integrated evaluation of two physiologically relevant functional carbohydrate fractions across three commercially available barley forms under standardized household processing conditions. Exploratory factorial analysis, interaction effects and effect-size estimates provided additional insights into the independent and combined influences of soaking and household thermal processing compared with descriptive analyses alone [11,18,19]. The resulting preliminary processing suggestions may provide a basis for selecting household processing approaches according to the desired functional carbohydrate profile while recognizing that processing responses are likely influenced by the interaction between household processing conditions and barley form [15,29,30].

The findings should be interpreted as exploratory observations rather than direct evidence of physiological benefit. While extractable β-glucan and total resistant starch are widely recognized as important dietary components associated with metabolic health, the present investigation did not evaluate molecular weight changes, viscosity, starch digestibility, glycaemic response, fermentation characteristics or other biological outcomes [3,5,6,7,32]. Consequently, the implications of the observed processing-induced compositional changes for human health require confirmation through appropriately designed mechanistic and intervention studies [32,33,34]. The following sections discuss the potential mechanisms underlying the differential responses of extractable β-glucan and total resistant starch and place the present findings within the context of the existing literature.

### 4.2. Differential Response of Extractable β-Glucan

The present study suggests that household thermal processing influenced extractable β-glucan content in barley. The observed responses varied across barley form and on the specific combination of soaking and household thermal processing applied. Importantly, these observations should be interpreted as changes in extractability rather than changes in the total β-glucan content of the grain. β-Glucan is a structural polysaccharide localized primarily within the endosperm cell walls of barley [35,36]. Therefore, the observed changes are appropriately interpreted as changes in extractability and accessibility rather than reflecting new synthesis or substantial degradation under conventional household processing conditions [35,37]. Instead, processing alters the physicochemical environment of the grain, affecting the extent to which β-glucan becomes solubilized and subsequently recovered under standardized analytical extraction conditions. This mechanism is inferred from the existing literature on cell wall biochemistry [11,35,37]. Consequently, the observed differences most likely reflect processing-induced modifications in cell-wall integrity, hydration characteristics and polymer accessibility rather than absolute changes in β-glucan concentration [11,35,37].

Among the household thermal processing methods evaluated, pressure cooking was associated with the greatest changes in extractable β-glucan content in pearled barley and barley grits. Pressure cooking combines elevated temperatures with saturated steam under pressure, promoting rapid water penetration, extensive starch gelatinization and disruption of endosperm cellular architecture. These structural changes are likely to facilitate the release and solubilization of cell-wall β-glucan, thereby increasing its extractability during analysis [11,18,35,37]. Previous studies have similarly reported enhanced β-glucan extractability following hydrothermal processing. These changes may be attributed to increased hydration, disruption of the cell-wall matrix and improved diffusion of soluble polysaccharides from the cereal endosperm [11,12,18,35]. At the same time, excessive thermal severity has been associated with depolymerization and reductions in molecular weight. These changes may influence viscosity and physiological functionality, even when the total β-glucan content remains relatively stable [7,31,37]. The moderate household processing conditions employed in the present study may therefore have favoured increased extractability without substantial degradation of the polymer. It is important to note that extractable β-glucan concentration alone does not determine its physiological functionality.

The influence of soaking on extractable β-glucan was smaller than that observed for total resistant starch, although significant effects were identified in all three barley forms. Soaking facilitates hydration of the grain and softening of the cellular matrix prior to cooking, thereby modifying heat and moisture transfer during subsequent household thermal processing [11,18,19]. However, the magnitude and direction of these effects varied according to barley form. These findings are consistent with the possibility that soaking may act primarily by modifying the physical accessibility of β-glucan, rather than directly altering its chemical structure [13,14,35]. The significant interaction observed in pearled barley and barley flour may indicate that the effects of soaking depend on the subsequent household thermal processing method rather than representing an independent processing factor.

Distinct responses were observed for barley flour compared with pearled barley and barley grits. Barley flour has a substantially smaller particle size and a greater degree of tissue disruption resulting from milling [13,14,35]. These characteristics increase the exposed surface area and facilitate rapid hydration during cooking, thereby altering the balance between β-glucan solubilization and potential thermal depolymerization [11,35,37,38]. Consequently, boiling following soaking was associated with the greatest extractable β-glucan in barley flour, whereas pressure cooking was associated with greater extractability in pearled barley and barley grits. These observations further support the importance of considering the form of the grain when evaluating processing-induced modifications in cereal carbohydrates. They further suggest that processing recommendations cannot be extrapolated across different barley forms, even when the same raw material is used [15,29,30].

Although household processing modified extractable β-glucan, the magnitude of these changes was generally smaller than those observed for total resistant starch, as reflected by the comparatively lower percentage changes and exploratory effect-size estimates. This difference is consistent with the distinct structural roles of these two carbohydrate fractions within the grain [3,5]. Whereas total resistant starch is considered to be highly susceptible to gelatinization and retrogradation during thermal processing, β-glucan appears to be influenced by changes in solubility and extractability [5,10,37,39]. Consequently, a household processing method that favours one functional carbohydrate fraction does not necessarily maximize the other. This further indicates the need for barley-form-specific household processing approaches, which may be preferable when different carbohydrate profiles are required. The mechanisms underlying the differential responses of total resistant starch are discussed in the following section.

### 4.3. Differential Response of Total Resistant Starch

The present study suggests that total resistant starch exhibited greater responsiveness to household processing than extractable β-glucan, as reflected by the consistently larger descriptive percentage changes and exploratory effect-size estimates observed across all three barley forms. This greater responsiveness may be consistent with the inherent physicochemical properties of starch, which could undergo extensive structural reorganization during hydrothermal processing [5,10,39,40]. While the observed changes in extractable β-glucan primarily reflect alterations in extractability, the total resistant starch content may be influenced by processing-induced modifications in starch architecture. These modifications could include gelatinization, molecular rearrangement and subsequent reassociation during cooling [5,10,39]. Consequently, total resistant starch might be sensitive to the combined effects of moisture, temperature and processing sequence, which make it a dynamic functional carbohydrate fraction during household preparation [5,19,39].

Pressure cooking was associated with the greatest increase in total resistant starch responses in pearled barley and barley grits. The combination of an elevated temperature, pressure and adequate moisture is likely to promote extensive starch gelatinization, increasing the mobility of amylose and amylopectin chains within the starch granules [10,39]. Upon cooling after cooking, these molecular chains may undergo reassociation, which could result in the formation of enzyme-resistant starch structures, likely including retrograded starch (RS3), which is widely recognized as the principal form generated during hydrothermal processing of cereal grains [5,39,40]. The greater total resistant starch contents observed following pressure cooking are consistent with conditions likely to promote starch reorganization during cooling rather than the formation of new starch components. This inference is made from the established starch chemistry literature in which similar mechanisms have been reported for cereal grains and other starch-rich foods, where hydrothermal processing followed by cooling was observed to increase resistant starch content through retrogradation of gelatinized starch [17,19,39,41]. The enzymatic method used in this study (AOAC 2002.02) quantifies total resistant starch and does not distinguish among resistant starch subtypes (RS1–RS4); hence, retrograded starch RS3 is proposed as the most likely contributing mechanism based on established starch chemistry. However, contributions from other resistant starch fractions cannot be excluded and warrant further investigation.

The influence of soaking further highlights the importance of processing sequence in determining total resistant starch responses. Although this could not be directly verified, soaking is likely to facilitate grain hydration before cooking and promote uniform heat transfer and starch gelatinization during subsequent household thermal processing, as reported by the literature [10,19]. Soaking alone may not substantially modify total resistant starch, but its interaction with household thermal processing could alter the extent of starch restructuring during cooling [19,41,42]. This interpretation is consistent with the significant soaking × household thermal processing interactions observed across all three barley forms. These findings are consistent with the possibility that soaking primarily functions as a preparatory step that modifies the effectiveness of subsequent thermal treatment, rather than acting as an independent determinant of total resistant starch content.

Distinct response patterns were observed in barley flour compared with pearled barley and barley grits for total resistant starch as well. Milling might disrupt the native grain architecture, reduce particle size and increase the exposed surface area available for hydration and heat transfer [13,14]. These structural modifications are likely to alter starch accessibility and gelatinization behaviour, potentially influencing the balance between starch disruption and subsequent molecular reassociation during cooling [10,16,38,39]. Consequently, barley flour exhibited a response profile in which the relative effectiveness of boiling depended on soaking status, whereas pressure cooking consistently produced the greatest total resistant starch responses in the other barley forms. These findings suggest that barley form may be an important determinant of processing-induced changes in total resistant starch and should be considered when developing household processing recommendations [15,29,30]. It should be noted here that total resistant starch content was analysed in cooked samples once they had cooled naturally to room temperature, without assisted, forced or refrigerated cooling. The extent to which the observed total resistant starch reflects RS3 formation is inferred from the previous literature rather than direct demonstration, as the specific resistant starch types were not quantified.

The greater responsiveness of total resistant starch compared with extractable β-glucan may have important implications for improving the functional carbohydrate profile of barley. β-glucan responses may primarily reflect changes in solubility and analytical extractability, and total resistant starch may be associated with structural reorganization within the starch matrix during processing [3,5,37,39]. This distinction may help explain why processing conditions that favour one functional carbohydrate fraction do not necessarily maximize the other. Collectively, these exploratory findings suggest that household processing strategies may need to consider both the desired functional carbohydrate profile and the specific barley form, rather than relying on a single processing strategy for all barley products. The broader implications of these barley-form-specific responses are discussed in the following section.

### 4.4. Influence of Barley Form and Household Processing on Functional Carbohydrate Responses

The response of functional carbohydrate fractions to household processing appeared to vary not only by the household thermal processing method itself but also by the processed barley form. Although identical soaking and household thermal processing conditions were applied across all treatments, the responses of extractable β-glucan and total resistant starch differed consistently among pearled barley, barley grits and barley flour. These findings suggest that the barley form may be an important determinant of processing-induced compositional changes and indicate that household processing methods may not be generalized across commercially available barley forms [15,29,30].

The contrasting responses observed among the barley forms may be attributable to differences in grain structure, particle size and the extent of tissue disruption resulting from commercial processing. Pearled barley largely retains its intact kernel architecture despite removal of the outer bran layers, whereas barley grits consist of fractured kernels with a greater exposed surface area, and barley flour represents the most extensively disrupted matrix following milling [13,14,35,36]. These structural differences may influence water absorption, heat transfer, starch gelatinization and the accessibility of cell-wall polysaccharides during household processing, thereby modifying the physicochemical environment in which β-glucan extractability and total resistant starch formation occur [10,16,30,37]. Consequently, identical household processing conditions may result in different functional carbohydrate responses depending on the barley form [15,29,30].

Beyond the influence of the form of the grain, the present findings indicate the importance of considering household processing as a sequence of interconnected preparation steps rather than as isolated operations. Soaking modified the observed responses to subsequent household thermal processing in a barley-form-specific manner, suggesting that pre-treatment modifies the hydration characteristics of the grain before thermal exposure [11,18,19]. This concept is supported by the significant interaction effects observed for most treatment combinations and highlights that the effectiveness of a household thermal processing method should not be interpreted independently of the preceding soaking step. Accordingly, these findings suggest that household processing methods may benefit from a complete preparation sequence rather than individual processing methods in isolation.

The use of standardized household processing conditions across three commercially available barley forms enabled a direct comparison of form-specific processing responses while minimizing methodological variation. This experimental approach facilitated the identification of barley-form-specific processing patterns that may not be evident when individual barley products are investigated separately. Moreover, the exploratory analytical framework, including factorial ANOVA, interaction effects, effect-size estimates and quantitative response patterns, may provide a framework for interpreting processing-induced modifications in functional carbohydrate composition [11,18,19].

Taken together, these findings suggest that the processed barley form may be an important determinant of the compositional response to household processing, providing a basis for the barley-form-specific processing patterns observed in the present study. Rather than reflecting the household processing method alone, functional carbohydrate responses appeared to be influenced by both processing conditions and the barley form [15,29,30]. Consequently, these exploratory findings may provide a basis for developing evidence-based, barley-form-specific household processing guidance, while requiring validation in larger and independent studies before broader application.

### 4.5. Translational Implications for Household Processing

The present findings have important practical implications for the household preparation of commercially available barley products. Although barley is widely recognized as an important source of functional carbohydrates, household processing recommendations are frequently generalized across different barley products without considering differences in grain structure or degree of processing [2,3,5]. The present study suggests that this approach may not maximize the functional carbohydrate profile, as identical household processing conditions produced distinct responses in pearled barley, barley grits and barley flour. Consequently, the selection of household processing methods may benefit from accounting for the structural characteristics of the specific barley form being prepared [15,29,30].

This study provides a preliminary framework for selecting household processing approaches according to the desired functional carbohydrate profile while recognizing the inherent differences among barley forms. The suggestions summarized in Table 4 are based on the recognition that household processing responses appeared to be barley-form-specific and therefore should account for differences in the structural characteristics of commercially available barley products. Accordingly, these suggestions should be interpreted as guidance derived from the compositional responses observed under standardized household preparation conditions rather than as universal recommendations applicable to all cereal products or processing environments. Instead, they provide a preliminary scientific basis for barley-form-specific household processing based on the interaction between processing conditions and food matrix characteristics that need to be validated further [15,29,30,33]. Furthermore, it should be noted that these recommendations are based only on compositional outcomes. Sensory attributes such as texture, taste, appearance, aroma, flavour, and mouthfeel, which may have an influence on consumer acceptability, were not evaluated in this study. As different cooking methods may affect these attributes differently depending on the intended food application, future studies should assess the sensory acceptability of barley products prepared using the recommended household processing methods.

This study also highlights the importance of considering household processing as a sequence of coordinated preparation steps. The interaction between soaking and household thermal processing indicates that pre-treatment can influence the effectiveness of subsequent cooking, emphasizing that household preparation may be better viewed as an integrated process rather than a series of independent operations [11,18,19]. This perspective may provide a basis for nutrition professionals, researchers and food scientists to interpret processing-induced modifications in the functional carbohydrate composition of barley prepared under household conditions and in designing future studies evaluating processing-related changes in cereal quality.

It is important to recognize that the present suggestions are based on compositional outcomes and do not directly infer physiological or clinical benefits. Although extractable β-glucan and total resistant starch are well-established functional carbohydrate components associated with favourable metabolic effects, the extent to which the observed processing-induced compositional changes influence viscosity, glycaemic response, colonic fermentation or other health outcomes remains to be determined [3,5,6,7,32]. Nevertheless, the present findings provide compositional evidence that may inform the future formulation and optimization of foods based on commercially available barley forms, while also informing the development of barley-form-specific household processing guidance [33]. Future investigations integrating compositional, physicochemical and physiological assessments, including well-designed human intervention studies, will be valuable for establishing whether the observed processing-induced compositional differences translate into clinically meaningful metabolic benefits [32,33,34].

### 4.6. Strengths, Limitations and Future Research Priorities

The present study possesses several strengths that may enhance the reliability and translational relevance of the findings. First, the effects of soaking and multiple household thermal processing methods were systematically evaluated using a factorial experimental design across three commercially available barley forms under standardized household preparation conditions. This design enabled an assessment of the independent and interactive effects of soaking and household thermal processing while facilitating a direct comparison of processing responses among barley forms. Second, this study employed validated AOAC-approved analytical procedures together with standardized enzymatic assays, moisture correction and effect-size analysis, providing a standardized methodological framework for accurately evaluating processing-induced changes in extractable β-glucan and total resistant starch [21,22,24,26,43,44]. Third, the simultaneous evaluation of two physiologically relevant functional carbohydrate fractions, namely extractable β-glucan and total resistant starch, provided a more comprehensive understanding of processing-induced compositional changes than studies focusing on a single carbohydrate component [3,5]. Finally, the integration of exploratory analysis, factorial ANOVA, interaction analyses, effect-size estimation and quantitative percentage-change synthesis through the heat map and preliminary household thermal processing suggestions facilitated the interpretation of the findings and may foster translation of the compositional data into practical, barley-form-specific household processing guidance.

Notwithstanding these strengths, several limitations should be acknowledged. This study evaluated compositional responses under controlled household processing conditions and did not investigate the physicochemical characteristics of the extracted β-glucan, including molecular weight distribution, viscosity or solubility, all of which may influence physiological functionality, as these were beyond the scope of the current study [6,7,31,37]. Similarly, although total resistant starch content was quantified, the specific resistant starch fractions were not characterized, and the mechanisms underlying the observed processing responses were inferred from established starch chemistry rather than directly measured [5,40]. Furthermore, the cooling conditions applied in this study may pose a limitation. Samples were cooled to ambient temperature without forced or rapid cooling to reflect typical household practice. The present study focused on standardized household thermal cooking methods representative of domestic practices and did not evaluate further processing variations, such as varying cooking durations and cooling conditions, as well as reheating and repeated cooking, all of which may further influence the functional carbohydrate profile of barley [19,39,41]. Future research should systematically investigate the influence of cooling time and temperature combinations on the formation and distribution of total resistant starch and its individual fractions (RS1–RS4). In addition, the present investigation did not evaluate starch digestibility, glycaemic response, colonic fermentation, sensory characteristics, consumer acceptability or other biological outcomes [6,32,34]. Consequently, the findings should be interpreted as indicative of compositional modulation rather than direct evidence of functional or clinical benefit. The comparatively large F-statistics and effect sizes observed reflect within-treatment variability under the experimental conditions evaluated. These effect-size implications are only indicative of narrow variations within batch analysis and do not constitute full confirmation of the reproducibility or a direct measure of biological effect. Their precise magnitude and biological effect should be confirmed through future studies using a larger sample size and independent household thermal processing and biological replicates. Therefore, the statistical findings of this study are presented as exploratory and hypothesis generating, requiring further validations with independent processing replicates.

A soaked-raw treatment was not included in the experimental design; consequently, the isolated effect of soaking, independent of subsequent cooking, was not measured in the present study. This effect should be examined in future studies to determine the extent of soaking’s contribution on its own. Likewise, only three commercially available barley forms were examined, and the findings may not be directly applicable to other barley cultivars or industrially processed products with different structural characteristics [13,14,38].

Furthermore, endpoints of household thermal processing methods were based on sensory criteria, consistent with household practices which may induce some subjectivity. Further studies that include instrumental parameters such as final internal temperatures, water absorption index or texture analysis may enhance the reproducibility of findings across laboratories. Another limitation of this study is that each treatment combination was processed only once, and the resulting statistical analyses represent within-batch variations and not a confirmation of reproducibility across independently processed samples. Replication was based on technical replicates from the same processed batch rather than on independently prepared batches. Future studies should include multiple independently prepared processing batches to confirm the reproducibility of these findings, although this was beyond the scope of the present study.

Future investigations should also build upon these findings by integrating compositional, physicochemical and physiological assessments to better understand the biological significance of processing-induced changes in functional carbohydrate composition. A characterization of β-glucan molecular weight distribution, viscosity and solubility, together with detailed resistant starch profiling, would provide greater mechanistic insights into the effects of household processing [6,7,31,37,40]. Controlled human intervention studies evaluating glycaemic response, satiety and colonic fermentation would help establish the physiological relevance of the processing strategies identified in the present study [6,32,34].

In addition, the evidence generated here may provide a preliminary basis for the future development of barley-based food formulations and household preparation strategies designed to tailor functional carbohydrate composition, although such applications require dedicated validation that is beyond the scope of the present investigation [33]. Future studies may also investigate the influence of household processing on additional quality attributes, including protein digestibility, polyphenol bio-accessibility and the interactions between functional carbohydrate composition and other bioactive constituents of barley [2,4,33]. Accordingly, the current findings should be viewed as hypothesis generating and, pending confirmation from larger studies, incorporating independent processing replicates, additional barley varieties and biological validation.

## 5. Conclusions

The present study suggests that the effects of household processing on extractable β-glucan and total resistant starch are dependent on the barley form and may not be generalized across commercially available barley products. Although the same household processing methods were applied under standardized conditions, pearled barley, barley grits and barley flour exhibited distinct functional carbohydrate responses, highlighting the importance of considering the barley form when interpreting processing-induced compositional changes. These findings suggest that household processing guidance needs to be tailored to the specific barley form rather than applying a single approach across all barley products.

Among the household thermal processing methods evaluated, pressure cooking was associated with the greatest observed potential to favourably modify the overall functional carbohydrate responses in pearled barley and barley grits, whereas barley flour exhibited a distinct processing profile, in which boiling resulted in greater extractability of β-glucan, and the relative influence of boiling for total resistant starch depended on soaking status. These observations suggest that tailoring household processing requires consideration of both the desired functional carbohydrate profile and the barley form.

The present investigation provides a preliminary framework for barley-form-specific household processing of barley based on the integrated evaluation of extractable β-glucan and total resistant starch under standardized household preparation conditions. By identifying processing approaches associated with greater extractable β-glucan or total resistant starch according to the barley form, the findings may assist future research investigating household processing approaches intended to improve the functional carbohydrate profile of barley. The findings also provide a preliminary basis for refining household processing of commercially available barley forms and may inform the future formulation and modifications of barley-based food products with an improved functional carbohydrate composition. Given the well-established physiological roles of β-glucan and total resistant starch, these processing-informed approaches may ultimately contribute to future dietary interventions and food-based strategies relevant to metabolic health, although confirmation of such benefits requires dedicated physicochemical, physiological and clinical investigations.

This study evaluated compositional responses under standardized household processing conditions and therefore provides a foundation for future research integrating physicochemical characterization, structural analyses and physiological validation. Further investigations incorporating β-glucan molecular characterization, resistant starch profiling, starch digestibility, glycaemic response, gut microbial fermentation and well-designed human intervention studies will be valuable for determining whether the processing-induced compositional changes identified in the present study translate into meaningful physiological and metabolic health benefits.

Collectively, the findings offer a preliminary scientific basis for barley-form-specific household processing strategies and suggest that improvements in functional carbohydrate profiles require consideration of both the household processing method and the structural characteristics of the processed barley form. This work provides a platform for future mechanistic, physiological and translational research and may inform the future formulation and modifications of barley-based food products through barley-form-specific processing approaches.

## Figures and Tables

**Table 1:** Standardized soaking conditions and household thermal processing established during preliminary optimization for 50 g of raw samples of pearled barley, barley grits and barley flour.

Processing Condition	Water Volume/Cooking Time	Non-Soaked			Soaked		
		Pearled Barley	Barley Grits	Barley Flour	Pearled Barley	Barley Grits	Barley Flour
Pre-treatment							
Soaking	Water (mL)	NA	NA	NA	200	200	500
	Time (h)	NA	NA	NA	15	2	2
Household thermal processing							
Pressure cooking	Water (mL)	500	250	600	350	200	500
	Time (min)	30	12	7	12	8	7
Microwave cooking	Water (mL)	800	350	600	800	350	500
	Time (min)	40	23	5	40	23	5
Boiling	Water (mL)	800	400	600	650	400	500
	Time (min)	47	15	6	38	6	6
Dry roasting	Water (mL)	NA	NA	NA	NA	NA	NA
	Time (min)	5	3	1	8	5	12

NA, not applicable. Cooking conditions were established during preliminary standardization experiments to achieve predefined cooking endpoints for each barley form.

**Table 2:** Extractable β-glucan content (g/100 g dry weight) of raw and household thermally processed pearled barley, barley grits and barley flour following non-soaking and soaking pre-treatment.

Household Thermal Processing Method	Pearled Barley		Barley Grits		Barley Flour	
	Non-Soaked	Soaked	Non-Soaked	Soaked	Non-Soaked	Soaked
Raw (reference)	4.24 ± 0.17 ^B^ (4.22–4.95)		5.31 ± 0.27 ^A^ (4.88–5.75)		4.59 ± 0.23 ^B^ (4.21–4.96)	
Pressure cooking	6.40 ± 0.22 ^a^ (6.05–6.75)	5.20 ± 0.07 ^a^ (5.09–5.31)	5.80 ± 0.17 ^a^ (5.53–6.07)	5.49 ± 0.29 ^a^ (5.03–5.95)	4.73 ± 0.04 ^a^ (4.67–4.79)	5.17 ± 0.06 ^a^ (5.07–5.27)
Microwave cooking	5.13 ± 0.01 ^b^ (5.11–5.15)	5.20 ± 0.23 ^a^ (4.83–5.57)	5.74 ± 0.24 ^a^ (5.36–6.12)	5.47 ± 0.16 ^a,b^ (5.22–5.72)	4.71 ± 0.16 ^a^ (4.46–4.96)	5.09 ± 0.16 ^a^ (4.84–5.34)
Boiling	5.08 ± 0.09 ^b^ (4.94–5.22)	4.85 ± 0.09 ^a^ (4.71–4.99)	5.60 ± 0.22 ^a^ (5.25–5.95)	5.12 ± 0.10 ^b,c^ (4.96–5.28)	4.75 ± 0.03 ^a^ (4.70–4.80)	5.20 ± 0.17 ^a^ (4.93–5.47)
Dry roasting	5.05 ± 0.07 ^b^ (4.94–5.16)	5.02 ± 0.44 ^a^ (4.32–5.72)	5.69 ± 0.10 ^a^ (5.53–5.85)	4.98 ± 0.06 ^c^ (4.88–5.08)	4.44 ± 0.10 ^b^ (4.28–4.60)	4.47 ± 0.04 ^b^ (4.41–4.53)

Values are presented as the mean ± SD (95% CI) of *n* = 4 technical replicate values. Raw values with different uppercase superscript letters differ significantly among barley forms (*p* < 0.05). Within each barley form and soaking condition, values with different lowercase superscript letters differ significantly among household thermal processing methods (Bonferroni-adjusted, *p* < 0.05).

**Table 3:** Total resistant starch content (g/100 g dry weight) of raw and household thermally processed pearled barley, barley grits and barley flour following non-soaking and soaking pre-treatment.

Household Thermal Processing Method	Pearled Barley		Barley Grits		Barley Flour	
	Non-Soaked	Soaked	Non-Soaked	Soaked	Non-Soaked	Soaked
Raw (reference)	5.24 ± 0.04 ^A^ (5.14–5.33)		2.10 ± 0.13 ^B^ (1.78–2.42)		0.22 ± 0.01 ^C^ (0.21–0.23)	
Pressure cooking	2.45 ± 0.05 ^a^ (2.33–2.57)	8.02 ± 0.11 ^a^ (7.75–8.29)	2.71 ± 0.07 ^a^ (2.54–2.88)	5.33 ± 0.09 ^a^ (5.11–5.55)	0.71 ± 0.02 ^b^ (0.66–0.76)	0.36 ± 0.02 ^d^ (0.31–0.41)
Microwave cooking	2.05 ± 0.02 ^b^ (2.00–2.10)	6.89 ± 0.44 ^b^ (5.80–7.98)	2.64 ± 0.19 ^a^ (2.17–3.11)	4.23 ± 0.10 ^b^ (3.98–4.48)	0.50 ± 0.02 ^b^ (0.45–0.55)	0.43 ± 0.00 ^c^ (0.43–0.43)
Boiling	1.74 ± 0.09 ^c^ (1.52–1.96)	4.76 ± 0.27 ^c^ (4.09–5.43)	1.94 ± 0.05 ^b^ (1.82–2.06)	4.22 ± 0.03 ^b^ (4.15–4.29)	1.07 ± 0.04 ^a^ (0.97–1.17)	0.49 ± 0.01 ^b^ (0.47–0.51)
Dry roasting	0.41 ± 0.02 ^d^ (0.36–0.46)	1.77 ± 0.05 ^d^ (1.59–1.83)	0.78 ± 0.04 ^c^ (0.68–0.88)	1.75 ± 0.08 ^c^ (1.55–1.95)	0.63 ± 0.02 ^b^ (0.58–0.68)	0.92 ± 0.03 ^a^ (0.85–0.99)

Values are presented as the mean ± SD (95% CI) of *n* = 3 technical replicate values. Raw values with different uppercase superscript letters differ significantly among barley forms (*p* < 0.05). Within each barley form and soaking condition, values with different lowercase superscript letters differ significantly among household thermal processing methods (Bonferroni-adjusted, *p* < 0.05).

**Table 4:** Potential household processing strategies suggested by extractable β-glucan and total resistant starch responses across commercially available barley forms.

Barley Form	Household Processing Associated with Greater Extractable β-Glucan	Household Processing Associated with Greater Total Resistant Starch	Potential Household Processing Approach
Pearled barley	Pressure cooking without soaking	Pressure cooking after soaking	Pressure cooking is the potential household processing approach. Whether soaking is included depends on the functional carbohydrate of interest.
Barley grits	Pressure cooking without soaking	Pressure cooking after soaking	Pressure cooking is the potential household processing approach. Whether soaking is included depends on the functional carbohydrate of interest.
Barley flour	Boiling after soaking	Boiling without soaking	Boiling is the potential household processing approach. Whether soaking is included depends on the functional carbohydrate of interest.

Recommendations are based on the integrated interpretation of extractable β-glucan and total resistant starch responses under the standardized household processing conditions evaluated in the present study. Household processing refers to the combination of soaking (where applicable) and the subsequent household thermal processing method.

## Data Availability

The original contributions presented in this study are included in the article/Supplementary Materials. Further inquiries can be directed to the corresponding author.

## Supplementary Materials

The following supporting information can be downloaded at: https://www.mdpi.com/article/10.3390/foods15173089/s1, Supplementary Table S1: Percentage moisture content of household thermal processing of barley forms and cooking methods for extractable β-Glucan and total resistant starch content; Supplementary Figure S1: Distribution of extractable β-glucan and total resistant starch content across barley forms and household thermal processing conditions; Supplementary Table S2: ANOVA assumption diagnostics by barley form for extractable β-Glucan and total resistant starch content; Supplementary Table S3: Exploratory two-way factorial ANOVA evaluating the effects of soaking, household thermal processing method and their interaction on extractable β-glucan and resistant starch in the three barley forms; Supplementary Table S4: Exploratory partial eta squared (η^2^p) effect-size estimates for soaking, household thermal processing method and their interaction for extractable β-glucan and resistant starch.
