# Supplementary material for: Differential Effects of Household Thermal Processing on Extractable β-Glucan and Total Resistant Starch Across Processed Barley Forms
Source: Foods. 2026 Aug 31;15(17):3089. doi: 10.3390/foods15173089 (PMC13564649; doi:10.3390/foods15173089)
Supplement: Supplementary file 1 [file foods-15-03089-s001.zip › foods-4461127-supplementary.pdf]

## Supplementary tables and figures

**Supplementary Table S1: Percentage moisture content of household thermal processing of barley forms and cooking methods for extractable β-Glucan and total resistant starch content**

| Household thermal processing method | Pearled barley | | Barley grits | | Barley flour | |
|---|---|---|---|---|---|---|
| | Non-soaked | Soaked | Non-soaked | Soaked | Non-soaked | Soaked |
| **A. Extractable β-Glucan content** | | | | | | |
| Pressure cooking | 85.66 | 85.70 | 84.13 | 83.23 | 85.59 | 83.66 |
| Microwave cooking | 80.32 | 75.26 | 71.88 | 75.55 | 84.18 | 86.10 |
| Boiling | 85.54 | 82.02 | 85.42 | 82.70 | 82.72 | 81.70 |
| Dry roasting | 5.63 | 6.00 | 18.26 | 28.41 | 10.85 | 71.44 |
| **B. Total resistant starch** | | | | | | |
| Pressure cooking | 84.76 | 84.88 | 83.02 | 81.96 | 84.40 | 82.42 |
| Microwave cooking | 79.06 | 73.72 | 70.18 | 73.73 | 83.30 | 84.71 |
| Boiling | 84.67 | 81.03 | 84.44 | 81.29 | 81.82 | 80.48 |
| Dry roasting | 2.22 | 2.38 | 13.64 | 24.39 | 5.49 | 69.46 |

*Values represent moisture content g/100g cooked sample) of the household thermally processed samples for each cooking method and barley form. Moisture content of β-Glucan reflects moisture associated with the processed samples and the residual moisture of the dried sample used for analysis. Moisture content of total resistant starch reflects the moisture present in the cooked sample as analysed.*

**Supplementary Figure S1: Distribution of extractable β-glucan and total resistant starch content across barley forms and household thermal processing conditions**

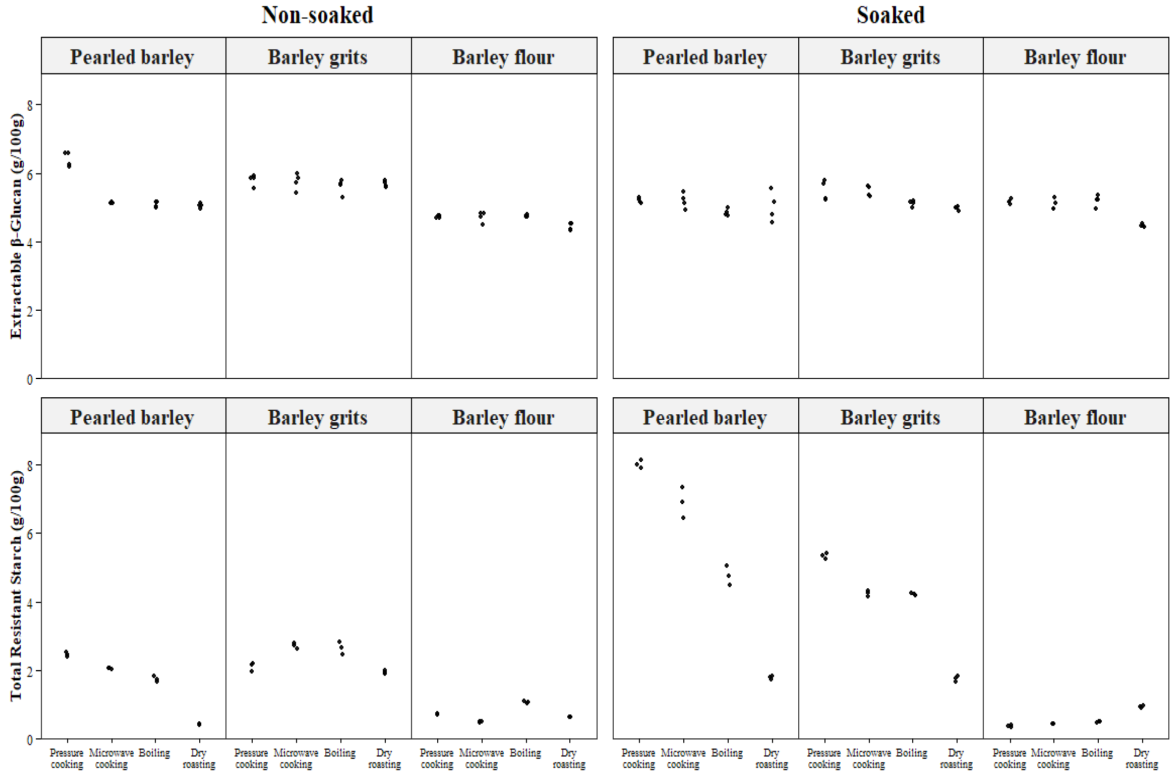


*Individual replicate values for extractable β-glucan (top) and total resistant starch content (below) of non-soaked and soaked groups across pearled barley, barley grits and barley flour samples and household thermal processing methods. Each dot represents a single replicate value (n=4 for extractable β-glucan content; n=3 for total resistant starch content) and is provided for visual representation of the data points underlying the mean values reported in Tables 2 and 3 of the manuscript.*

**Supplementary Table S2: ANOVA assumption diagnostics by barley form for extractable β-Glucan and total resistant starch content**

| Barley form | Shapiro–Wilk W | p-value | Normality | Levene's F | p-value | Homogeneity |
|---|---|---|---|---|---|---|
| **A. Extractable β-Glucan** | | | | | | |
| Pearled barley | 0.945 | 0.106 | Satisfied | 7.193 | <0.001 | Violated |
| Barley grits | 0.958 | 0.234 | Satisfied | 1.890 | 0.116 | Satisfied |
| Barley flour | 0.962 | 0.307 | Satisfied | 1.377 | 0.260 | Satisfied |
| **B. Total Resistant starch** | | | | | | |
| Pearled barley | 0.849 | 0.002 | Violated | 2.320 | 0.077 | Satisfied |
| Barley grits | 0.936 | 0.136 | Satisfied | 1.591 | 0.208 | Satisfied |
| Barley flour | 0.618 | <0.001 | Violated | 3.291 | 0.023 | Violated |

*Given the balanced design, the ANOVA was considered robust to modest violations observed in main effects.*

**Supplementary Table S3. Exploratory two-way factorial ANOVA evaluating the effects of soaking, household thermal processing method and their interaction on extractable β-glucan and resistant starch in the three barley forms.**

| Barley form | Source of variation | df | F | p-value |
|---|---|---|---|---|
| **A. Extractable β-glucan** | | | | |
| Pearled barley | Soaking | 1 | 24.530 | <0.001 |
| | Cooking method | 3 | 29.140 | <0.001 |
| | Soaking × Cooking | 3 | 16.806 | <0.001 |
| Barley grits | Soaking | 1 | 46.494 | <0.001 |
| | Cooking method | 3 | 6.167 | 0.003 |
| | Soaking × Cooking | 3 | 2.398 | 0.093 |
| Barley flour | Soaking | 1 | 69.043 | <0.001 |
| | Cooking method | 3 | 40.692 | <0.001 |
| | Soaking × Cooking | 3 | 6.755 | 0.002 |
| **B. Total Resistant Starch** | | | | |
| Pearled barley | Soaking | 1 | 2252.848 | <0.001 |
| | Cooking method | 3 | 539.892 | <0.001 |
| | Soaking × Cooking | 3 | 148.074 | <0.001 |
| Barley grits | Soaking | 1 | 2361.805 | <0.001 |
| | Cooking method | 3 | 958.818 | <0.001 |
| | Soaking × Cooking | 3 | 92.794 | <0.001 |
| Barley flour | Soaking | 1 | 329.313 | <0.001 |
| | Cooking method | 3 | 275.217 | <0.001 |
| | Soaking × Cooking | 3 | 369.230 | <0.001 |

*Two-way factorial ANOVA was performed separately for each barley form to evaluate the main effects of household pre-processing (non-soaked and soaked), household thermal processing (pressure cooking, microwave cooking, boiling and dry roasting) and their interaction on extractable β-glucan and resistant starch. Degrees of freedom (df), F-statistics (F) and corresponding p-values are presented. Statistical significance was accepted at $p < 0.05$.*

*Raw samples served as baseline reference values and were not included in the factorial ANOVA because they were not part of the factorial experimental design.*

**Supplementary Table S4. Exploratory partial eta squared ($\eta^2p$) effect-size estimates for soaking, household thermal processing method and their interaction for extractable β-glucan and resistant starch.**

| Barley form | Source of variation | Partial $\eta^2$ | 95% CI |
|---|---|---|---|
| **A. Extractable β-glucan** | | | |
| Pearled barley | Soaking | 0.505 | 0.208-0.691 |
| | Cooking method | 0.785 | 0.590-0.867 |
| | Soaking × Cooking | 0.678 | 0.407-0.798 |
| Barley grits | Soaking | 0.660 | 0.405-0.791 |
| | Cooking method | 0.435 | 0.090-0.630 |
| | Soaking × Cooking | 0.231 | 0.000-0.455 |
| Barley flour | Soaking | 0.742 | 0.532-0.842 |
| | Cooking method | 0.836 | 0.684-0.899 |
| | Soaking × Cooking | 0.458 | 0.112-0.647 |
| **B. Resistant Starch** | | | |
| Pearled barley | Soaking | 0.993 | 0.984-0.996 |
| | Cooking method | 0.990 | 0.995-0.997 |
| | Soaking × Cooking | 0.965 | 0.919-0.981 |
| Barley grits | Soaking | 0.993 | 0.984-0.996 |
| | Cooking method | 0.994 | 0.987-0.997 |
| | Soaking × Cooking | 0.946 | 0.874-0.970 |
| Barley flour | Soaking | 0.954 | 0.894-0.974 |
| | Cooking method | 0.981 | 0.956-0.989 |
| | Soaking × Cooking | 0.986 | 0.967-0.992 |

*Partial eta squared ($\eta^2p$) values were calculated from the two-way factorial ANOVA to estimate the proportion of variance associated with soaking, household thermal processing and their interaction. In this exploratory study, $\eta^2p$ values along with p-values were used to describe the relative magnitude of the observed effects.*

*As the analyses were based on technical replicates from single processed batches, these effect sizes are exploratory and should not be considered direct measures of biological effect size or reproducibility.*
